# Diffusion-weighted MRI in the evaluation of the thyroid nodule: Comparison between integrated-shimming EPI and conventional 3D-shimming EPI techniques

**DOI:** 10.18632/oncotarget.25279

**Published:** 2018-05-25

**Authors:** Luguang Chen, Peipei Sun, Qiang Hao, Wei Yin, Bing Xu, Chao Ma, Alto Stemmer, Caixia Fu, Minjie Wang, Jianping Lu

**Affiliations:** ^1^ Department of Radiology, Changhai Hospital of Shanghai, Shanghai, China; ^2^ MR Application Development, Siemens Healthcare GmbH, Erlangen, Germany; ^3^ MR Application Development, Siemens Shenzhen Magnetic Resonance Ltd, Shenzhen, China

**Keywords:** diffusion-weighted imaging, thyroid nodule, single-shot echo planar imaging, integrated-shimming echo planar imaging, dynamic shimming

## Abstract

This study aimed to evaluate whether a prototype echo planar imaging sequence with integrated-shimming (iShim-EPI) can improve image quality in the thyroid gland in comparison to 3D-volume shimming echo planar imaging (3D-Shim-EPI), and to compare ADC values derived from iShim-EPI with those of 3D-Shim-EPI. Twenty-one patients with thyroid disease were enrolled and underwent axial DWIs with iShim-EPI and 3D-Shim-EPI using a 3 Tesla magnetic resonance scanner in this prospective study. Both sets of DWI images were evaluated by two independent observers who identified susceptibility and ghost artifacts and evaluated the images' capacity to detect thyroid nodules using quantitative scores. The ADC values of the thyroid nodules and the normal thyroid gland were measured two times within a 4-week period. The reproducibility was evaluated using the intraclass correlation coefficient (ICC) and Bland-Altman plots. There were significant differences in the image quality scores for susceptibility (2.81 ± 0.37 vs. 1.93 ± 0.29, *p* < 0.001), ghost artifacts (2.95 ± 0.15 vs. 1.93 ± 0.29, *p* < 0.001) and the detectability of thyroid nodules (3.00 ± 0.00 vs. 2.55 ± 0.75, *p* = 0.008) between the iShim-EPI and 3D-Shim-EPI techniques, except for the ADC values of the thyroid nodules (1.607 ± 0.466×10^−3^ mm^2^/s vs. 1.561 ± 0.483 × 10^−3^ mm^2^/s, *p* = 0.184) and contralateral normal thyroid gland (1.295 ± 0.340 × 10^−3^ mm^2^/s vs.1.279 ± 0.411 × 10^−3^ mm^2^/s, *p* = 0.777). Both techniques demonstrated excellent agreement between the ADC values using the ICC (range, 0.963 to 0.999) and Bland-Altman plots. The iShim-EPI technique demonstrated significantly higher image quality compared with the conventional 3D-Shim-EPI technique, with no significant differences in the ADC values.

## INTRODUCTION

The thyroid nodule is a common disorder of the thyroid gland [1]. The prevalence is highest in females. The overall incidence of thyroid nodules in the general population is 4–7%, of which 5–7% possess the potential for malignancy [2, 3]. Although a small proportion of thyroid nodules are malignant, early and accurate diagnosis of the malignant thyroid is crucial for effective treatment of these patients.

Several imaging modalities, including ultrasonography, computed tomography, radionuclide scintigraphy and positron emission tomography, have been used to distinguish malignant thyroid nodules from benign ones. However, these imaging techniques can have several drawbacks, including ionizing radiation, poor accuracy and subsequent limited utility [4]. With the advancement of imaging technologies, magnetic resonance imaging (MRI) is becoming a useful tool to evaluate thyroid gland disease due to its noninvasiveness and better image contrast compared with other imaging techniques. The routine MRI (including T1-Weighted, T2-Weighted and contrast enhanced T1-Weighted imaging) has been used to assess the extent of the disease; however, it remains difficult to accurately predict the nature of thyroid nodules using these structural images [5, 6].

Diffusion-weighted imaging (DWI) is a noninvasive method for evaluating Brownian motion of microscopic water diffusion in tissues *in vivo*, and this technique has been widely used in routine examinations. A variety of studies have reported that the apparent diffusion coefficient (ADC) values derived from diffusion-weighted images can be used to discriminate between benign and malignant thyroid nodules [1, 7–9]. However, image distortions caused by susceptibility changes and ghost artifacts, for example, severely degrade the image quality of DWI in the neck region, especially poor at 3 Tesla (T) field strength.

For DWI, single-shot echo planar imaging is the most commonly used method due to its relative insensitivity to motion-induced phase errors, its high signal-to-noise ratio (SNR) and short acquisition time [10]. However, EPI images are normally obscured by blurring along the phase-encoding direction because of T2* decay and are sensitive to off-resonance effects [11], particularly in the neck region. Distortions in EPI (Δd(r)) are unavoidable and are proportional to the phase field of view (FOV_PE_), the echo spacing (Δt_PE_) and the local off-resonance (ΔB_0_) [12]. Therefore, the distortions can be expressed as Δd(r) ~ FOV_PE_ * Δt_PE_ * ΔB_0_. Several techniques have been developed to reduce EPI distortions. For instance, parallel imaging and reduced FOV methods with two-dimensional radio frequency-excitation (2DRF-excitation) (e.g. the ZOOMit technique) have been used to reduce the FOV_PE_, and the readout-segmented EPI decreases the Δt_PE_ [11, 13, 14]. Recently, a novel technique called integrated-shimming echo planar imaging (iShim-EPI), combining integrated slice-specific dynamic shimming and pixel-wise unwrapping distortion correction, has been proposed to reduce distortions and signal voids caused by local B_0_ inhomogeneity [15]. Previous studies [12, 16] have demonstrated how iShim-EPI outperformed 3D-Shim-EPI on diffusion imaging through distortion reduction and lesion detection in the neck region.

In this study, we hypothesized that iShim-EPI could be used for DWI in the thyroid gland, which is prone to severe artifacts in the neck region. To the best of our knowledge, no such study for the thyroid gland has been reported in the literature. Therefore, the goals of this study were to evaluate whether iShim-EPI can improve the image quality of the thyroid gland DWI in comparison to 3D-Shim-EPI, and to compare the ADC values, acquired from iShim-EPI with those of 3D-Shim-EPI.

## RESULTS

All 21 patients included in this study underwent MRI scans. However, three patients were excluded from the quantitative analysis because of poor image quality (scores were = 1). The histologic assessment revealed 8 patients had papillary thyroid carcinoma, 2 patients had medullary thyroid carcinoma, 6 patients had nodular goiter and 2 patients had thyroid adenoma.

### Qualitative comparisons of image quality

**Figure 1:**
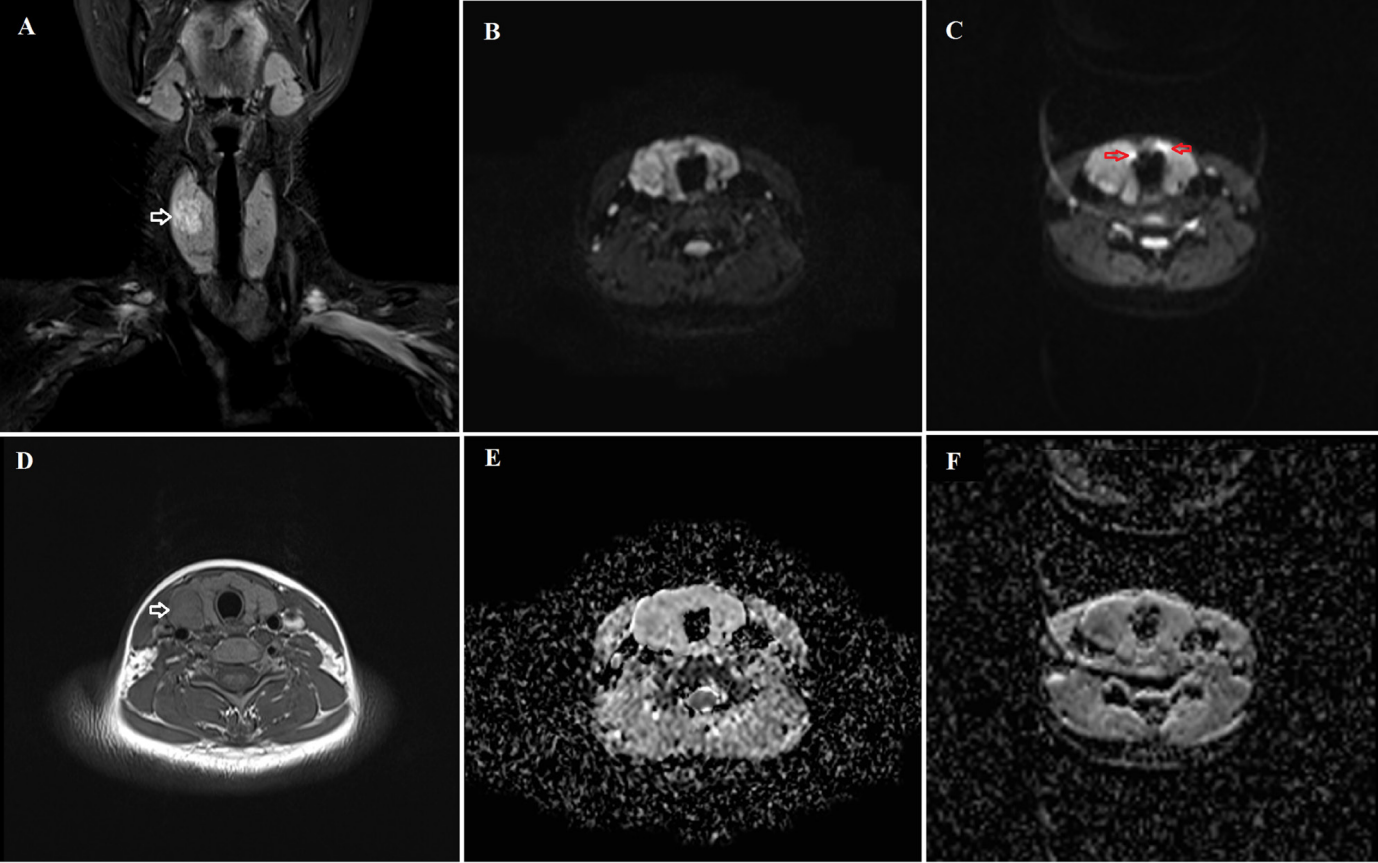
Comparison of the iShim-EPI and 3D-Shim-EPI sequences in a 26-year-old female with a thyroid nodule in the right thyroid lobe (**A**) is a T2-weighted image with fat saturation and (**D**) is a T1-weighted image. Hyperintensity and hypointensity characteristics of the thyroid nodule were observed on these images (white arrows). The iShim-EPI DWI image had b = 500 s/mm^2^ (**B**) and ADC map (**E**). 3D-Shim-EPI DWI image with b = 500 s/mm^2^ (**C**) and ADC map (**F**). Susceptibility (red arrows) and ghost artifacts are shown on the 3D-Shim-EPI images, whereas improved image quality was observed using the iShim-EPI technique.

**Figure 2:**
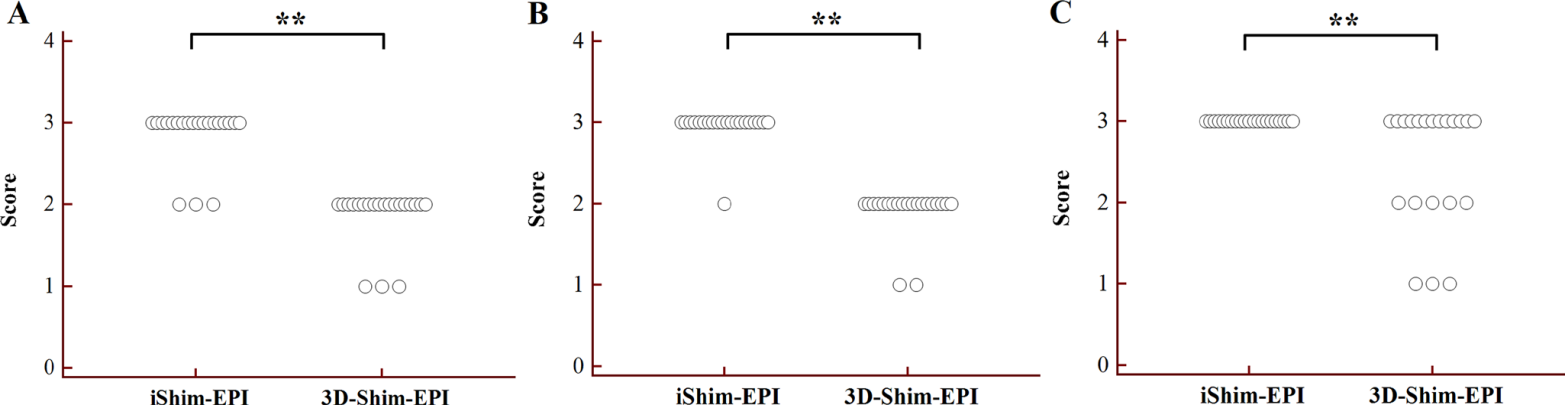
Dot plots of susceptibility (**A**), ghost artifacts (**B**) and detectability of thyroid nodules (**C**) for the iShim-EPI and 3D-Shim-EPI techniques. ^**^P < 0.005.

The analysis results of image quality for the iShim-EPI and 3D-Shim-EPI techniques are shown in Table 1. Figure 1 shows example images with iShim-EPI and 3D-Shim-EPI images from a patient with thyroid nodules. For both Observers 1 and 2, the average scores for iShim-EPI were significantly higher than those with 3D-Shim EPI images for susceptibility artifacts (2.81 ± 0.37 vs. 1.93 ± 0.29, *p* < 0.001), ghost artifacts (2.95 ± 0.15 vs. 1.93 ± 0.29, *p* < 0.001), and the detectability of thyroid nodules (3.00 ± 0.00 vs. 2.55 ± 0.75, *p* = 0.008) (Figure 2), respectively.

**Table 1:** Comparison of qualitative scores for thyroid nodules for the iShim-EPI and 3D-Shim-EPI techniques

Observers	Protocols	Susceptibility	Ghost artifacts	Detectability
Observer 1	iShim-EPI	2.86 ± 0.36	2.95 ± 0.22	3.00 ± 0.00
	3D-Shim-EPI	1.86 ± 0.36	1.90 ± 0.30	2.48 ± 0.75
	*p value*	<0.001	<0.001	0.009
Observer 2	iShim-EPI	2.76 ± 0.44	2.95 ± 0.22	3.00 ± 0.00
	3D-Shim-EPI	2.00 ± 0.32	1.95 ± 0.38	2.62 ± 0.74
	*p value*	<0.001	<0.001	0.038
Observers 1 & 2	iShim-EPI	2.81 ± 0.37	2.95 ± 0.15	3.00 ± 0.00
	3D-Shim-EPI	1.93 ± 0.29	1.93 ± 0.29	2.55 ± 0.72
	*p value*	<0.001	<0.001	0.008

*Note*: Data are expressed for the two observers as Observer 1 and Observer 2. Values show the mean ± standard deviation for image quality.

### Quantitative comparisons of ADC values

**Figure 3:**
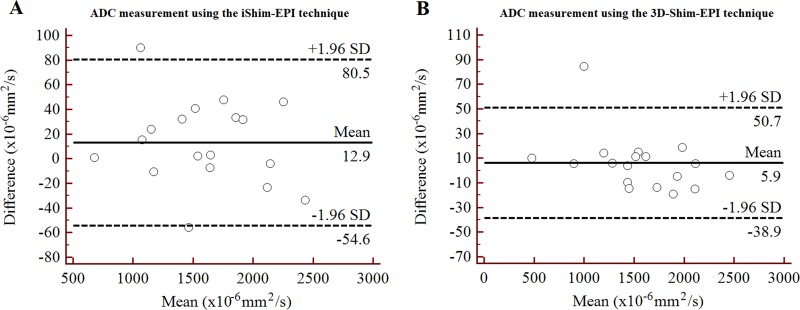
Bland-Altman plots of ADC measurements using the iShim-EPI (**A**) and 3D-Shim-EPI (**B**) techniques in patients with thyroid nodules.

**Figure 4:**
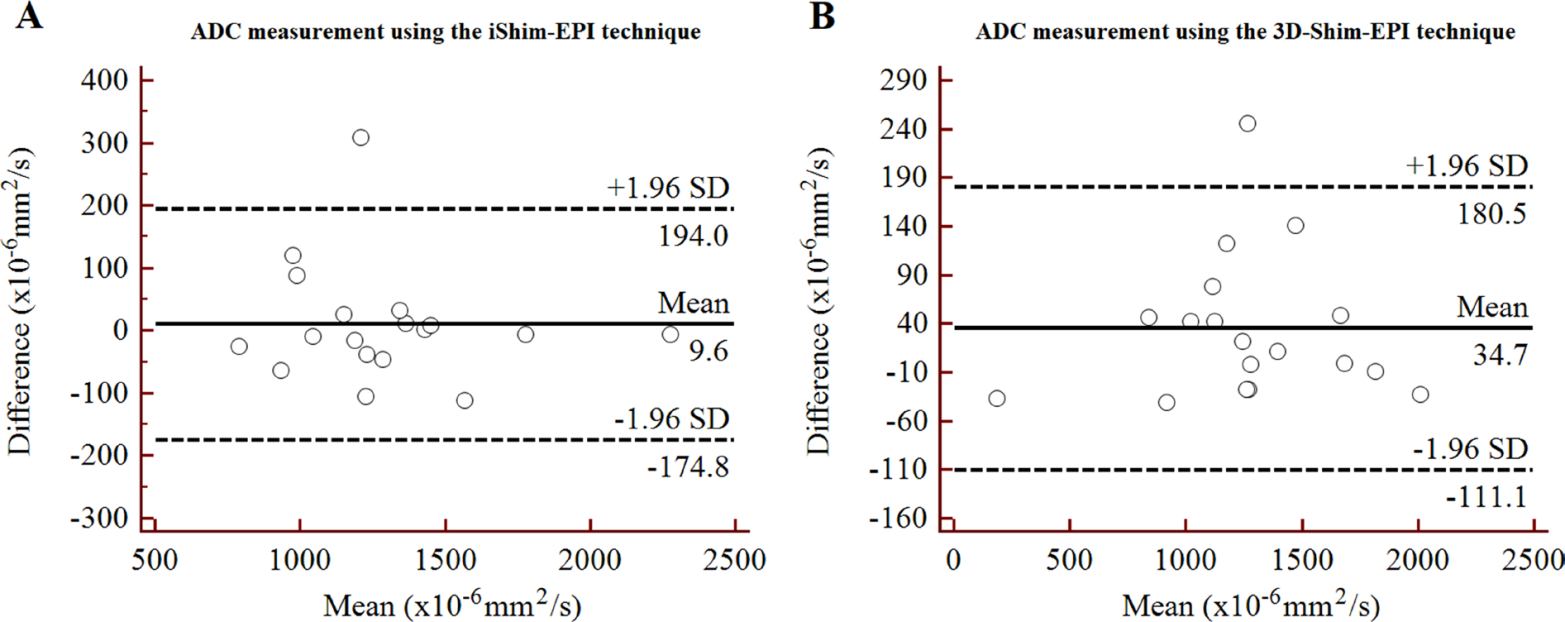
Bland-Altman plots of ADC measurements using the iShim-EPI (**A**) and 3D-Shim-EPI (**B**) techniques in patients with contralateral normal thyroid gland.

There was no significant difference between the iShim-EPI and 3D-Shim-EPI sequences for the ADC values of the thyroid nodules (1.607 ± 0.466 × 10^−3^ mm^2^/s vs. 1.561 ± 0.483 × 10^−3^ mm^2^/s, *p* = 0.184), as well as the contralateral normal thyroid gland (1.295 ± 0.340 × 10^−3^ mm^2^/s vs. 1.279 ± 0.411 × 10^−3^ mm^2^/s, *p* = 0.777). The reproducibility of the ADC measurements is summarized in Table 2. In addition, an excellent agreement was demonstrated for the ADC values of the thyroid nodules and contralateral normal tissues employing the iShim-EPI and 3D-Shim-EPI sequences, with ICCs ranging from 0.963 to 0.999. The Bland-Altman plots also showed excellent reproducibility of the ADC measurements using both techniques for the evaluation of the thyroid nodules (Figure 3) and contralateral normal thyroid gland (Figure 4).

**Table 2:** Quantitative comparison of ADC measurement between the iShim-EPI and 3D-Shim-EPI techniques in patients with thyroid nodules and contralateral normal thyroid gland

Tissues	Protocols	Measurement 1 (mean ± SD)	Measurement 2 (mean ± SD)	*p* value	ICC (95% CI)
Lesion	iShim-EPI	1607.51 ± 466.30	1594.60 ± 472.82	0.157	0.997 (0.993–0.999)
	3D-Shim-EPI	1561.37 ± 483.65	1555.49 ± 492.97	0.42	0.999 (0.997–0.999)
Normal	iShim-EPI	1295.22 ± 340.57	1285.59 ± 353.98	0.777	0.963 (0.905–0.986)
	3D-Shim-EPI	1279.51 ± 411.45	1244.80 ± 409.11	0.085	0.984 (0.957–0.994)

*Note*: Data are listed for the two measurements as Measurement 1 and Measurement 2. SD, standard deviation. ICC, Intra-class coefficient, CI, confidence interval.

## DISCUSSION

In this study, we demonstrated a much higher image quality of thyroid DWI using iShim-EPI compared with the conventional 3D-Shim-EPI technique. Image quality of iShim-EPI with respect to susceptibility artifacts, ghost artifacts and the detectability of thyroid nodules was superior to that using the 3D-Shim-EPI technique. However, there was no significant difference in the ADC values obtained using both techniques. In addition, an excellent reproducibility was demonstrated for the ADC measurements using the iShim-EPI and 3D-Shim-EPI techniques for evaluating thyroid nodules and normal tissue.

As one of the most commonly used functional MRI, DWI has emerged as an important method that can be noninvasively evaluate the diffusion of microscopic water molecules through tissues. DWI was initially used to evaluate brain disease, such as stroke, which offered better sensitivity and specificity for the evaluation of ischemia [17, 18]. Furthermore, DWI has been extensively used to detect and assess some diseases in many other organs in the past several years, including, for example, liver carcinoma, pancreatic cancer, renal carcinoma, prostate cancer, rectal cancer and cervical cancer [19–25]. DWI can also be used to differentiate benign tumors from malignant ones using ADC values. Of note, 3D-Shim-EPI has conventionally been used to evaluate disease in clinical practice because of both its resistance to ghost artifacts and fast acquisition time. Several studies have been reported to use this technique for assessing thyroid lesions [7–9, 11, 26–29]. However, it is very challenging to perform conventional DWI in the neck region where the local field inhomogeneity cannot be completely compensated by shimming and therefore can lead to signal voids and image distortions. In this study, the 3D-Shim-EPI and iShim-EPI techniques were used on thyroid nodule DWI and the results compared. The iShim-EPI technique uses a slice-specific dynamic shimming scheme rather than 3D volume shimming of the entire stack of slices. This appears to remarkably improve the magnetic field homogeneity of each slice. Additionally, the pixel-vise unwrapping distortion correction method was used to remove any residual distortion due to the second or higher order field inhomogeneity. Thus, artifacts were significantly reduced in the iShim-EPI images. To our knowledge, the application of iShim-EPI in the evaluation of thyroid nodules has thus far not been explored.

In our study, the image quality scores of the iShim-EPI images were found to be higher than those of the 3D-Shim-EPI images in respect to susceptibility artifacts, ghost artifacts and the detectability of thyroid nodules. The image quality scores benefitted from the slice-specific dynamic shimming scheme combined with the pixel-vise unwrapping distortion method. Gatidis *et al.* showed that the utility of iShim-EPI for DWI contributes to improved image quality in the neck region at 3 T compared with other EPI-based techniques (3D-Shim-EPI, Zoomed EPI and readout-segmented EPI) [12]. Zhang *et al.* demonstrated that iShim-EPI can improve lesion detection in whole-body DWI at 3 T [16]. Similar results were consistently demonstrated in our study.

The ADC values of the thyroid nodules and contralateral normal thyroid gland were evaluated quantitatively for both techniques. No significant differences in the ADC measurements using the 3D-Shim-EPI and iShim-EPI techniques were observed (both had *p* values > 0.05). However, the average ADC values of the thyroid nodules and contralateral normal thyroid gland on iShim-EPI were higher than those on 3D-Shim-EPI. The reason may be due to better shimming using iShim-EPI compared with 3D-Shim-EPI, thus less susceptibility and distortion were observed on the iShim-EPI images, which would slightly vary the ADC values. Additionally, we used the shortest repetition time/echo time (TR/TE) for both sequences respectively, while kept the other parameters consistent. Several studies have shown that ADC values depend on various imaging parameters, such as magnetic field strength, TR, TE, the b value and gradient mode, which might explain why ADC values are slightly different between the two DWI sequences [30–32]. We also assessed the reproducibility of ADC measurements on lesions and normal tissues. Figures 3 and 4 show the excellent reproducibility of the ADC values using Bland-Altman plots, and narrow intervals of agreement compared with the mean were observed for these measurements. Additionally, we also noted an excellent agreement of these measurements using the ICC values. The consistency of ADC values in both techniques and improved image quality revealed that we may use the new iShim-EPI technique to replace conventional 3D-Shim-EPI technique for routine thyroid DWI examination.

The present study has several limitations. First, the patient population was relatively small. A larger patient population is needed to verify our findings and for further study. Second, iShim-EPI can decrease but not thoroughly eliminate susceptibility and distortion artifacts. Therefore, a more advanced technique within the sphere of MRI technology is needed to overcome these artifacts. Third, iShim-EPI and 3D-Shim EPI techniques were performed with their shortest TR/TE respectively. As discussed above, different TR/TE might influence the calculation of the ADC value, however, no significant differences were observed in ADC between both techniques in the present study. But further study with strictly identical parameters is conducted. Finally, the benign and malignant properties of thyroid nodules were not differentiated by the ADC values. Since this was not the main purpose of the present study, we will evaluate this feature with a larger sample size in later studies.

In conclusion, iShim-EPI can decrease reduce susceptibility and ghost artifacts, improve the overall image quality and the detectability of thyroid nodules compared with conventional 3D-Shim-EPI. Notably, iShim-EPI DWI may serve in clinical practice as a promising technique for decreasing artifact and assessing thyroid disease.

## MATERIALS AND METHODS

### Subjects

Between October 2015 and July 2016, 21 patients (age, 22–84 years; male/female, 4/17) with thyroid nodules were consecutively enrolled in this prospective study. The inclusion criterion was patients who were found to have thyroid nodules diagnosed by ultrasound. Thyroid nodules of diameter less than 5 millimeters, with ill-defined borders and of poor image quality were excluded from the study [29]. This study was approved by the local institutional review board, and written informed consent was obtained from each patient.

### Image acquisition

Magnetic resonance imaging was performed on a 3 T whole body MRI system (MAGNETOM Skyra, Siemens Healthcare, Erlangen, Germany) using a 20-channel phased-array head/neck coil. The thyroid gland examination protocols included an axial T1-weighted turbo spin-echo (T1W TSE), coronal T2-weighted TSE and axial DWI sequences using both a prototype iShim-EPI and 3D-Shim-EPI techniques with a acquisition time of 1minute 17 seconds and 1minute 16 seconds, respectively. T1W and T2W images were used to locate thyroid nodules and position the DWI sequences. The parameters of these protocols are summarized in Table 3. The total scan time of each subject was approximately seven minutes.

**Table 3:** MRI protocols and main parameters of thyroid gland examination

Protocols	TR/TE (ms)	FOV (mm^2^)	Matrix	Slice number	Thickness (mm)	Gap (mm)	b value (s/mm^2^)	Averages	Scan time
Axial T1W TSE	550/12	240 × 240	224 × 320	20	4	0.4	–	3	2:07
Cor T2W FS TSE	3000/83	240 × 240	218 × 256	13	4	0.4	–	2	2:29
Axial iShim-EPI	2200/75	240 × 240	136 × 136	14	4	0.0	50, 500	4	1:17
Axial 3D-Shim-EPI	2400/56	240 × 240	136 × 136	14	4	0.0	50, 500	4	1:16

*Note*: TSE, turbo spin echo; TR/TE, repetition time / echo time; FOV, field of view; FS, fat saturation; iShim-EPI, integrated-shimming echo planar imaging; 3D-Shim-EPI, 3D-volume shimming echo planar imaging.

### Image analysis

All morphological images and DWI images were transferred to a workstation (syngoMMWP, Siemens Healthcare, Germany) for analysis. iShim-EPI and 3D-Shim-EPI DWI images were evaluated by two independent observers (Observer 1 and Observer 2 with four and six years of experience in thyroid imaging, respectively) who were blind to the subjects' clinical information, with T1W and T2W images serving as a reference to identify susceptibility artifacts, ghost artifacts (1, marked; 2, median; 3, no artifact), and the detectability of thyroid nodules (1, poor; 2, median; 3, good) using three-scale scores. Freehand circular regions of interest (ROIs) were drawn in the nodule and normal regions of the thyroid gland on ADC maps by the first Observer, cautiously avoiding vessels, and areas of necrosis and hemorrhage. The mean value of the ADC was recorded for each ROI. To test the intra-observer variability, the observer measured the two sets of ADC images twice with an intervening 4-week period to avoid any recall bias.

### Statistical analysis

All the analyses were performed using SPSS (version 16.0, SPSS Inc., Chicago, IL, USA) and MedCalc (version 13.0.0.0, MedCalc Softaware, Mariakerke, Belgium) softwares for Microsoft Windows. Quantitative data were described as mean ± standard deviation. Significant differences in image quality scores, and ADC values between iShim-EPI and 3D-Shim-EPI were assessed using Wilcoxon signed-rank tests. The intra-observer variability was evaluated using the intraclass correlation coefficient (ICC) and Bland-Altman plots for ADC values [33]. ICC values < 0.4, 0.4 – 0.75 and > 0.75 were regarded as representing poor agreement, good agreement and excellent agreement, respectively [34]. A two-tailed *p* value less than 0.05 was deemed to represent statistical significance.

## Abbreviations

3D-Shim-EPI: 3D-volume shimming EPI
ADC: Apparent diffusion coefficient
DWI: Diffusion-weighted imaging
FOV_PE_: Phase field of view
ICC: Intraclass correlation coefficient
iShim-EPI: Integrated-shimming echo planar imaging
MRI: Magnetic resonance imaging
ROI: Region of interest
SNR: Signal-to-noise ratio
TSE: Turbo spin-echo
ΔB_0_: Local off-resonance
Δd(r): Distortions in EPI
Δt_PE_: Echo spacing
